# A Systematic Scoping Review on Pedagogical Strategies of Interprofessional Communication for Physicians in Emergency Medicine

**DOI:** 10.1177/23821205211041794

**Published:** 2021-10-16

**Authors:** Zhi H. Ong, Lorraine H. E. Tan, Haziratul Z. B. Ghazali, Yun T. Ong, Jeffrey W. H. Koh, Rachel Z. E. Ang, Chermaine Bok, Min Chiam, Alexia S. I. Lee, Annelissa M. C. Chin, Jamie X. Zhou, Gene W. H. Chan, Gayathri D. Nadarajan, Lalit K. R. Krishna

**Affiliations:** 1 Yong Loo Lin School of Medicine, National University of Singapore, Singapore; 2 Division of Supportive and Palliative Care, National Cancer Centre Singapore, Singapore; 3 National University of Singapore, Singapore; 4 Alice Lee Centre for Nursing Studies, National University of Singapore, Singapore; 5 Division of Cancer Education, National Cancer Centre Singapore, Singapore; 6 Medical Library, National University of Singapore Libraries, Singapore; 7 Lien Centre for Palliative Care, Duke-NUS Medical School, Singapore; 8 Duke-NUS Medical School, Singapore; 9 National University Hospital, National University Health System, Singapore; 10 Singapore General Hospital, Singapore; 11 Palliative Care Institute Liverpool, Academic Palliative & End of Life Care Centre, UK; 12 Centre for Biomedical Ethics, National University of Singapore, Singapore; 13 PalC, The Palliative Care Centre for Excellence in Research and Education, Singapore

**Keywords:** emergency medicine, interprofessional, communication, medical education

## Abstract

**Background:**

Interprofessional communication (IPC) is integral to interprofessional teams working in the emergency medicine (EM) setting. Yet, the coronavirus disease 2019 pandemic has laid bare gaps in IPC knowledge, skills and attitudes. These experiences underscore the need to review how IPC is taught in EM.

**Purpose:**

A systematic scoping review is proposed to scrutinize accounts of IPC programs in EM.

**Methods:**

Krishna's Systematic Evidence-Based Approach (SEBA) is adopted to guide this systematic scoping review. Independent searches of ninedatabases (PubMed, Embase, CINAHL, Scopus, PsycINFO, ERIC, JSTOR, Google Scholar and OpenGrey) and “negotiated consensual validation” were used to identify articles published between January 1, 2000 and December 31, 2020. Three research teams reviewed the data using concurrent content and thematic analysis and independently summarized the included articles. The findings were scrutinized using SEBA's jigsaw perspective and funneling approach to provide a more holistic picture of the data.

**Results:**

**In total:**

18,809 titles and abstracts were identified after removal of duplicates, 76 full-text articles reviewed, and 19 full-text articles were analyzed. In total, four themes and categories were identified, namely: (a) indications and outcomes, (2) curriculum and assessment methods, (3) barriers, and (4) enablers.

**Conclusion:**

IPC training in EM should be longitudinal, competency- and stage-based, underlining the need for effective oversight by the host organization. It also suggests a role for portfolios and the importance of continuing support for physicians in EM as they hone their IPC skills.

**Highlights:**

• IPC training in EM is competency-based and organized around stages.

• IPC competencies build on prevailing knowledge and skills.

• Longitudinal support and holistic oversight necessitates a central role for the host organization.

• Longitudinal, robust, and adaptable assessment tools in the EM setting are necessary and may be supplemented by portfolio use.

## Introduction

Interprofessional communication (IPC) is defined as the “sharing of information among members of different health care professionals to influence patient care positively” - highlighting the role of effective communication and collaboration within teams of health care professionals including physicians, nurses, technicians, administrative staff, and community service providers.^1^ IPC enhances empathetic, sensitive and appropriate communications^2–2^ and facilitates history taking,^6^ team working,^7–7^ patient compliance,^10^ and clinical outcomes.^11–13^ Effective IPC also improves patient satisfaction,^14–18^ professional accountability and responsibility,^19^ better-informed consent and reduced medical errors and harm.^20–28^

Perhaps nowhere is the value of IPC more evident than in emergency medicine (EM). An academic speciality since the 1960s, IPC training is now an integral aspect of many EM training curricula. Yet, such development has not been consistent globally.^29^ Up to 2012, two-fifths of EU countries did not recognize EM as a specialty.^29, 30^ The impact of this delay has limited IPC training for staff working in EM's fast-paced,^21, 31–37^ stressful, high stakes,^11–13, 25^ overcrowded,^38^ and often disruptive setting.^21, 32^ The impact of these gaps in training are further underscored amidst growing public attention on bed crises, overcrowding, and mounting waiting times.^21, 39–42^

With a consistent approach to IPC training in EM continuing to elude practice,^43–46^ and guided by the importance placed upon it by the Accreditation Council for Graduate Medical Education (ACGME)^20, 23^ and the Institute of Medicine,^11, 25, 26, 47–50^ we seek to evaluate regnant accounts of IPC training in EM to attend to this gap in understanding^27, 51^ and guide efforts to design a setting specific training approach.^11, 52–55^

## Methods

To overcome concerns as to the transparency and reproducibility of systematic scoping reviews (SSR)s, Krishna's Systematic Evidence-Based Approach (SEBA) is adopted. SSRs in SEBA adopt a constructivist perspective enabling them to map a complex topic from multiple angles^56^ while a relativist lens helps account for the context-specific, sociocultural sensitive and user-dependent nature of IPC training.^57–60^

To provide a balanced review and enhance accountability, SSRs in SEBA undergo a 6-staged process. Each stage involves input from an expert team consisting of a medical librarian from the Yong Loo Lin School of Medicine (YLLSoM) at the National University of Singapore (NUS), clinicians specialized in EM at the National University Hospital and Singapore General Hospital, and local education experts and clinicians at the National Cancer Centre Singapore (NCCS), Palliative Care Institute Liverpool, YLLSoM, and Duke-NUS Medical School. The expert team fulfills several roles, such as reviewing, questioning, and challenging the findings of the research team to ensure a robust and evidence-based driven position as well as aiding with reflexivity and trustworthiness.^61, 62^

**Figure 1.**
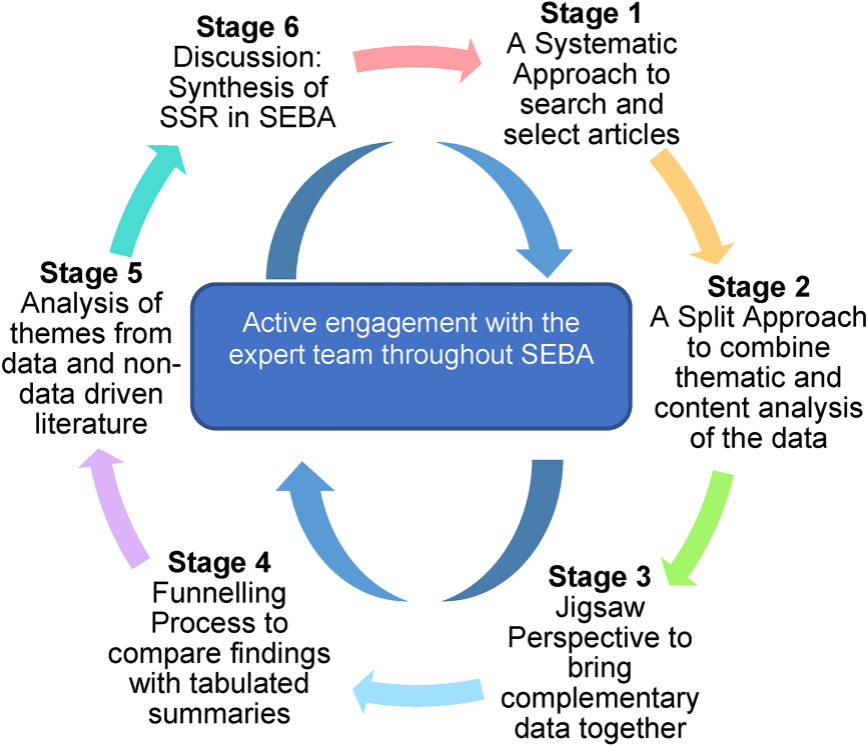
The Systematic Evidence-Based Approach (SEBA) process was employed to identify patterns and relationships among studies.

The research and expert teams adopt an interpretivist approach as they proceed through the 6 stages of SEBA^57–60^ (see Figure 1).

### Stage 1 of SEBA: Systematic Approach

#### Identifying the Research Question

Ensuring a systematic approach to the synthesis of SSRs in SEBA, the research and expert teams agreed upon the goals, population, context, and concept to be evaluated. The two teams then determined the primary research question to be *“what is known about IPC training for physicians in EM?”* and the secondary research question to be *“what are the characteristics of these IPC programs?”*

#### Inclusion Criteria

These questions were designed on the population, concept, and context elements of the inclusion and exclusion criteria,^63^ using a Population, Intervention, Comparison, Outcomes and Study (PICOS) format (Table 1).

**Table 1.** PICOS, inclusion criteria, and exclusion criteria applied to database search.

PICOS	Inclusion criteria	Exclusion criteria
Population	Postgraduate emergency medicine physicians and residents Nurses and allied health personnel including occupational therapist, physiotherapist and pharmacist, social workers, dieticians and nutritionist, and supporting clinical staff	Alternative medicine, nonmedical specialties (eg dentistry)
Intervention	Educational interventions for physicians about interprofessional communication through face-to-face or real-time virtual communication approaches	Evaluation tools on multidisciplinary communication Educational interventions that are ONLY for other members of health care team about interprofessional communication with physicians and do not involve physicians at all
Comparison	Comparison of educational strategies, assessment methods, outcomes measures and challenges in developing interprofessional communication	
Outcome	Strategies to develop interprofessional communication, assessment methods on the effectiveness of interventions Outcomes and challenges in developing interprofessional communication	
Study design	Articles in English or translated to English Variety of study designs not limited to mixed methods research, randomized controlled trials, cohort studies, case-control studies, cross-sectional studies, and descriptive papers Year of Publication: 1 January 2000 to 31 December 2020 Databases: PubMed, Embase, CINAHL, Scopus, PsycINFO, ERIC, JSTOR, and Google Scholar, OpenGrey	

#### Searching

Six members of the research team carried out independent searches of eight bibliographic databases (PubMed, Embase, Cumulative Index to Nursing and Allied HealthLiterature (CINAHL), Scopus, Psychological Information Database (PsycINFO), Education Resources Information Center (ERIC), Journal Storage (JSTOR), Google Scholar) and one gray literature database (OpenGrey). In keeping with Pham et al's ^64^ recommendations on ensuring a viable and sustainable research process, the research team confined the searches to articles published between January 1, 2000 to December 31, 2019 to account for prevailing manpower and time constraints. Additionally, another search was conducted on June 26, 2021 to update articles that were published from January 21, 2020 to December 31, 2020. The PubMed search strategy can be found in Supplemental Material A.

#### Study Selection and Data Charting

The research team independently screened the title and abstracts identified and created individual lists of titles to be included. These were discussed at online meetings. Consensus was achieved on the final articles to be included using Sandelowski, Barroso's^65^ “negotiated consensual validation” approach which sees *“research team members articulate, defend, and persuade others of the 'cogency' or 'incisiveness' of their points of view or show their willingness to abandon views that are no longer tenable"*. Eight members of the research team independently reviewed all the full-text articles featured on the final lists, discussed their individual lists at online meetings and reached an agreement once more. 

In keeping with the SEBA methodology, the research team then evaluated the references of the included articles. This “snowballing” of references was carried out to ensure a more comprehensive review.

### Stage 2 of SEBA: Split Approach

Three teams of researchers simultaneously and independently reviewed the included full-text articles. Here, the combination of independent reviews by various members of the research teams using different methods of analyses provided triangulation^66^ while detailing the analytical process improved audits and enhanced the authenticity of the research.^62^

The first team summarized and tabulated the included full-text articles in keeping with recommendations drawn from Wong et al's^67^ “RAMESES publication standards: meta-narrative reviews” and Popay et al's^56^ “Guidance on the conduct of narrative synthesis in systematic reviews.” The tabulated summaries ensured that key aspects of the included articles were not lost. These tabulated summaries also included quality appraisals using the Medical Education Research Study Quality Instrument^68^ and the consolidated criteria for reporting qualitative (COREQ)^69^ studies (Supplemental Material B).

Concurrently, the second team analyzed the included articles using Braun and Clarke's^70^ approach to thematic analysis. In Phase 1, the research team carried out independent reviews, actively reading the included articles to find meaning and patterns in the data.^72–76^ In Phase 2, “codes” were constructed from the “surface” meaning and collated into a codebook to code and analyze the rest of the articles using an iterative step-by-step process. As new codes emerged, these were associated with previous codes and concepts. In Phase 3, the categories were organized into themes that best depict the data. An inductive approach allowed themes to be “*defined from the raw data without any predetermined classification*”.^75^ In Phase 4, the themes were refined to best represent the whole data set. In Phase 5, the research team discussed the results of their independent analyses online and at reviewer meetings. “*Negotiated consensual validation*” was used to determine if saturation was achieved and establish the final themes. These involved members articulating and defending their analyses where discrepancies arose until consensus was reached.

The third team of researchers employed Hsieh and Shannon's^77^ approach to directed content analysis which involved “*identifying and operationalizing a priori coding categories*.”

The research team drew codes from Miller's^83^ treatise entitled “*The assessment of clinical skills/competence/performance*” to guide the coding of the articles. Any data not captured by these codes were assigned a new code. In keeping with deductive category application, coding categories were reviewed and revised as required.

Findings were then discussed online until consensus was reached. The final codes were compared and discussed with the final author who checked the primary data sources to ensure that the codes made sense and were consistently employed. Any differences in coding were resolved between the research team and the final author. “Negotiated consensual validation” was used as a means of peer debrief in allthree teams to further enhance the validity of the findings.^84^

The narrative produced was guided by the Best Evidence Medical Education Collaboration guide^85^ and the Structured approach to the Reporting In health care education of Evidence Synthesis statement.^86^

### Stage 3 of SEBA: Jigsaw Perspective

As part of SEBA's reiterative process, the themes and categories identified were discussed with the expert team. Here, the themes and categories are viewed as pieces of a jigsaw puzzle and areas of overlap allow these pieces to be combined to create a wider andholistic view of the overlying data. The combined themes and categories are referred to as themes/categories.

Creating themes/categories relied on the use of Phases 4 to 6 of France et al's ^88,89^ adaptation of Noblit and Hare's seven phases of meta-ethnography.^92^ Themes and categories were contextualized by reviewing them against primary codes and subcategories and/or subthemes they were drawn from.^87, 88^ Reciprocal translation was used to determine if the themes and categoriescould be used interchangeably.

### Stage 4 of SEBA: The Funnelling Process

The funneling process sees the themes/categories identified compared with the tabulated summaries.

To provide structure to the funneling process, we employed Phases 3 to 5 of France et al'sadaptation and described the nature, main findings and conclusions of the articles. 

Adapting Phase 5, reciprocal translation was used to juxtapose the themes/categories with key messages identified in the tabulated summaries. The verified themes/categories from the Funnelling Process then formed *“the line of argument”* in the discussion synthesis, in Stage 6 of the SSR in SEBA.

## Results

**Figure 2.**
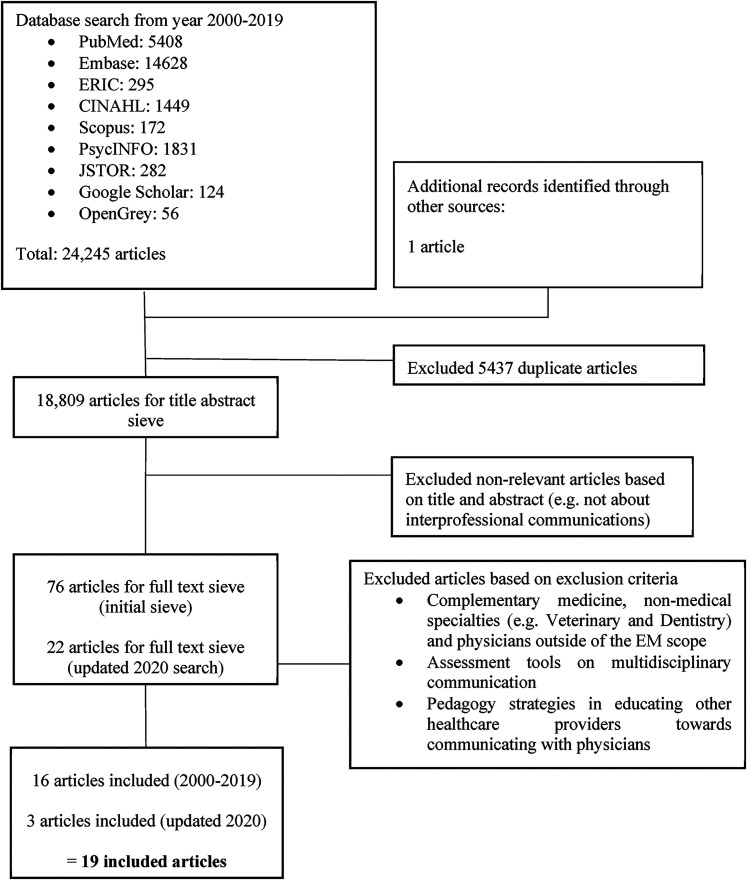
PRISMA flow chart.

In total, 18,809 titles and abstracts were identified after the removal of duplicates, 98 full-text articles reviewed, and 19 full-text articles analyzed (Figure 2: Preferred Reporting Items for Systematic Reviews and Meta-Analyses (PRISMA) flow chart).

### Themes/Categories

The final themes/categories identified were:

- Indications for IPC programs.
- Curriculum and assessment.
- Outcomes of IPC programs.
- Barriers to IPC programs in ED.
- Enablers for successful IPC programs.

#### Indications for IPC Programs

Given that these themes/categories were not discussed and often merely listed in the included articles, they are summarized in Table 2 for ease of review.

**Table 2.** Perceived role and indications in EM training.

Indications for the need for effective IPC	References
Failure leads to medical errors/poor patient outcomes	^25, 50, 92–101^
Intensity of EM setting	^20, 92–94, 96, 99, 100, 102^
Complex nature of the job as EM physician	^20, 52, 103^
Integral to delivering high-quality patient care/positive patient outcomes	^20, 99, 101, 104^

Abbreviations: EM, emergency medicine; IPC, interprofessional communication.

#### Curriculum and Assessment

IPC training often begins with didactic teaching that serve to introduce foundational knowledge in both core or elective-based curriculum content.^52, 92, 94, 96, 97, 105^ This new-found knowledge is then applied in high fidelity simulations with mannikins,^93, 96, 97^ virtual patients,^93^ and/or simulations in-situ.^95, 103^ Simulations improve team dynamics by providing a *“controlled, adaptable environment,”*^93^ that facilitates mutual understandingand changes in attitude, instils respect^96^ and overcomes “*social constructs of hierarchy*” and “*silo mentality*.”^93^

**Figure 3.**
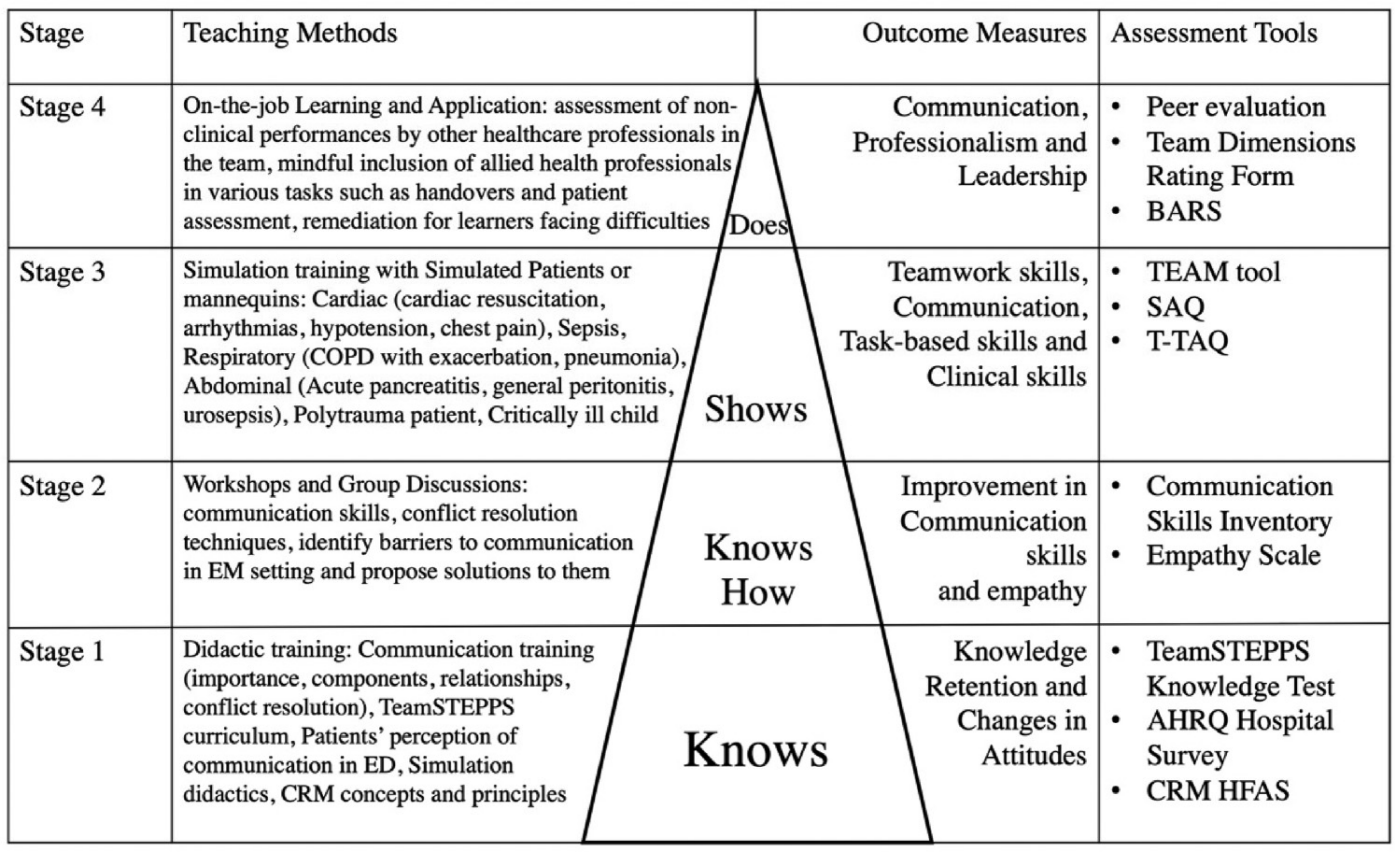
IPC in EM curriculum and assessmment mapped onto Miller's pyramid.

Abbreviations: AHRQ, Agency for Healthcare Research and Quality; BARS, behaviorally anchored rating scales; COPD, chronic obstructive pulmonary disease; CRM, crisis resource management; HFAS, Human Factors Attitude Survey; EM, emergency medicine; IPC, interprofessional communication; SAQ, short answer questions; T-TAQ, Teamwork Attitudes Questionnaire.

A gradation in training knowledge, skills and attitudes is summarized in Figure 3 built upon the four stages of Miller’s pyramid and complemented by a variety of assessment tools. These include the use of the Human Factors Attitude Survey to evaluate attitudinal shifts posttraining^106^, as well as the TeamSTEPPS Teamwork Attitude Questionnaire and other behaviorally anchored rating scales to assess teamwork.^50^

Each stage of the learning process is accompanied by outcome measures and assessment tools which are also summarized in Supplemental Material C.

#### Outcomes of IPC Programs

The outcome measures for effective IPC also take a long-term perspective as summarized in Table 3.

**Table 3.** Outcomes in EM IPC training.

Outcomes of IPC training	References
Host organization	
Improved host organization outcomes (hospital performance and reimbursement, patient safety outcomes)	^96–98, 102^
Reduction in adverse events and errors	^25, 50, 98, 100^
Liability cost savings	^50^
Participants and colleagues	
Improvements in attitudes and behavior towards IPC, teamwork, and internal communications	^52, 95–97, 101, 103, 104, 106^
Increased safety attitudes	^95, 103^
Patients	
Greater satisfaction in the quality of staff and patient communications	^52, 96^
Reduced communication issues between staff and patients	^52^
Reduced length of hospital stay	^97^
Improved patient experience	^52^

Abbreviations: EM, emergency medicine; IPC, interprofessional communication.

#### Barriers to IPC Programs in ED

The long-term nature of the goals of IPC programs in EM is reflected in the obstacles to achieving them.^92, 93^ These include frequent turnover of staff, staff of various backgrounds, skills, experience and clinical specialities, shift work, high patient load, rapid and complex decision-making and past negative interactions with other health care professionals.^20, 92^ In turn, these barriers hinder culture change, shifts in institutionalized power dynamics and flattening of the hierarchical nature within ED.^107^

#### Enablers for Successful IPC Programs

Enablers to a successful IPC program similarly reflect its long term goals and include the sustained support of the host organization,^96^ the provision of tutor training and compensation, mandating attendance, ensuring effective assessments of learning outcomes and effective provision of feedback.^96^

### Stage 5 of SEBA: Analysis of Themes From Data and Nondata Driven Literature

With concerns over the potential impact of unevidenced, often opinion-led data from gray literature, the research team undertook a comparison of themes identified from gray literature and those from research-driven articles to enhance the accountability and the reproducibility of Stage 6 of SEBA. The results from the gray literature were found to be congruent with those from peer-reviewed research-based articles.

## Discussion

### Stage 6 of SEBA: Synthesis of SSR in SEBA

In answering its primary research question on *“what is known about IPC training for physicians in EM?”* ,this SSR in SEBA of IPC training in EM suggests that IPC training occurs in stages. This begins with the use of didactic training that then progresses to the application of the physician’s new knowledge. Practice in the clinical situation follows, culminating in the formation of attitudes and behaviors that value IPC communications. Supporting this premise that IPC competencies develop on prevailing knowledge and skills is the presence of a variety of assessment tools aimed at assessing IPC skills and attitudes along with this longitudinal development.

Having established the presence of a longitudinal training and assessment process that corresponds with Miller’s Pyramid of Clinical Competence as shown in Figure 3, it is possible to deduce specific stages in the development of IPC skills. In addressing its secondary research question, this SSR in SEBA suggests that IPC programs in EM are characterized by competency-based stages in the training and development of IPC knowledge, skills and attitudes among EM physicians. The achievement of skills in each stage is assessed by the corresponding tools suggesting the presence of competency-based stages thatalign with the competencies set out by ACGME^20, 23^ and the Institute of Medicine.^11^ Such developments are also consistent with the stated outcomes of these programs and may also be inferred from the enablers and barriers to IPC programs.

Here, the presence of stages underscores the importance of the host organization in ensuring an effective assessment of the various stages, curricula topics and providing assiduous program oversight and support.^97^ This also underlines the need for training and assessments to be carried out at appropriate junctures in the development of EM trainees to ensure they achieve requisite competencies in core topics before venturing to secondary content.

This further highlights the need for the IPC training program specific assessment process to account for the personalized needs of varied learners.^108, 109^ To this end, portfolios may be suitable learning and evaluation tools for IPC in EM due to their flexible and holistic nature—allowing for multisource feedback, documentation and reflection of learning activities and assessment scores in one convenient central location. This would offer a comprehensive overview of the learners’ progress, enabling easy identification of areas for improvement.^110–112^

Overall, this SSR in SEBA highlights the need for greater awareness of the competency-based stages in the development of IPC skills and urges more holistic and longitudinal consideration of its effects upon the physician.

## Limitations

Despite efforts to enhance the reproducibility and transparency of the SSR through SEBA, we still face gaps in our methodology and analysis. While we have conducted a two-tiered searching strategy, through both independent searching of selected databases by our expert team and repeated sieving of reference lists of publications, there maybe important papers that have been omitted. Similarly, while use of the split approach and tabulated summaries in SEBA allowed for triangulation and ensured that a holistic picture was constructed from different and diverse perspectives, inherent biases among the reviewers may still have impacted the analysis of the data and construction of themes. Lastly, drawing conclusions from a limited pool of largely North American and European-centric accounts, further narrowed by focusing upon publications in English, may limit the applicability of the findings to other cultural and geographical contexts.

## Conclusion

Data on the stages of training forwarded by this SSR will be of interest to educationalists and program designers involved in IPC training in EM, where teamwork and communication are instrumental in the provision of safe and effective care for patients, especially in global health emergencies such as the coronavirus disease 2019 pandemic. A stage-based curriculum suggests that trainers need to be aware of the level of experience and abilities of physicians in training and cater their training approach and assessments to meet these personalized needs. Development of skills and abilities needed for effective IPC should begin in medical school and junior residency programs, underlining the role of portfolios to capture their competencies and reflections on their training thus far. Gaps in understanding and supporting trainers, guiding the host organization, and the lack of effective oversight of the stage-wise development of IPC competencies underline areas for further study if IPC training is to achieve its goals of improving safety and patient experience in EM.
